# Cystic Fibrosis Newborn Screening in Austria Using PAP and the Numeric Product of PAP and IRT Concentrations as Second-Tier Parameters

**DOI:** 10.3390/diagnostics11020299

**Published:** 2021-02-13

**Authors:** Maximilian Zeyda, Andrea Schanzer, Pavel Basek, Vera Bauer, Ernst Eber, Helmut Ellemunter, Margit Kallinger, Josef Riedler, Christina Thir, Franz Wadlegger, Angela Zacharasiewicz, Sabine Renner

**Affiliations:** 1 Clinical Division of Pediatric Pulmonology, Allergology and Endocrinology, Department of Pediatrics and Adolescent Medicine, Comprehensive Center for Pediatrics, Medical University of Vienna, 1090 Vienna, Austria; andrea.schanzer@meduniwien.ac.at (A.S.); sabine.renner@meduniwien.ac.at (S.R.); 2 University Clinic for Paediatric and Adolescent Medicine, University Hospital Salzburg, 5020 Salzburg, Austria; p.basek@salk.at; 3 Department of Pediatrics and Adolescent Medicine, Klinikum Wels-Grieskirchen, 4600 Wels, Austria; vera.bauer@klinikum-wegr.at; 4 Division of Pediatric Pulmonology and Allergology, Department of Pediatrics and Adolescent Medicine, Medical University of Graz, 8036 Graz, Austria; ernst.eber@medunigraz.at; 5 Department of Child and Adolescent Health, Division of Cardiology, Pulmonology, Allergology, and Cystic Fibrosis, Cystic Fibrosis Centre, Medical University of Innsbruck, 6020 Innsbruck, Austria; helmut.ellemunter@i-med.ac.at; 6 PEK Hospital Steyr Department of Pediatrics and Adolescent Medicine, 4400 Steyr, Austria; margit.kallinger@ooeg.at; 7 Department of Pediatrics and Adolescent Medicine, Kardinal Schwarzenberg Hospital Schwarzach, 5620 Schwarzach, Austria; josef.riedler@kh-schwarzach.at; 8 Department of Paediatrics and Adolescent Medicine, Johannes Kepler University Linz, 4040 Linz, Austria; christina.thir@kepleruniklinikum.at; 9 Division of Pediatric Pulmonology and Allergology, Department of Pediatrics and Adolescent Medicine, Hopital Klagenfurt am Wörthersee, 9020 Klagenfurt am Wörthersee, Austria; franz.wadlegger@lkh-klu.at; 10 Department of Pediatrics and Adolescent Medicine, Klinikum Ottakring, Wilhelminenspital, Teaching Hospital of the University of Vienna, 1010 Vienna, Austria; angela.zacharasiewicz@gesundheitsverbund.at; 11 Associated National Center in the European Reference Network for Rare Respiratory Diseases, ERN-LUNG Coordinating Center, University Hospital Frankfurt, 60596 Frankfurt am Main, Germany

**Keywords:** neonatal screening, recalls, false-positives, IRT×PAP, IRT-PAP

## Abstract

In Austria, newborns have been screened for cystic fibrosis (CF) by analyzing immunoreactive trypsinogen (IRT) from dried blood spots (DBS)s for nearly 20 years. Recently, pancreatitis-associated protein (PAP) analysis was introduced as a second-tier test with the aim of reducing recalls for second DBS cards while keeping sensitivity high. For 28 months, when IRT was elevated (65–130 ng/mL), PAP was measured from the first DBS (*n* = 198,927) with a two-step cut-off applied. For the last 12 months of the observation period (*n* = 85,421), an additional IRT×PAP cut-off was introduced. If PAP or IRT×PAP were above cut-off, a second card was analyzed for IRT and in case of elevated values identified as screen-positive. Above 130 ng/mL IRT in the first DBS, newborns were classified as screen-positive. IRT analysis of first DBS resulted in 1961 (1%) tests for PAP. In the first 16 months, 26 of 93 screen-positive were confirmed to have CF. Two false-negatives have been reported (sensitivity = 92.8%). Importantly, less than 30% of families compared to the previous IRT-IRT screening scheme had to be contacted causing distress. Adding IRT×PAP caused a marginally increased number of second cards and sweat tests to be requested during this period (15 and 3, respectively) compared to the initial IRT-PAP scheme. One case of confirmed CF was found due to IRT×PAP, demonstrating an increase in sensitivity. Thus, the relatively simple and economical algorithm presented here performs effectively and may be a useful model for inclusion of CF into NBS panels or modification of existing schemes.

## 1. Introduction

Cystic fibrosis (CF) is an autosomal recessive inherited disease characterized by accumulation of viscous secretion in the pancreas, lungs, and other organs, severely impairing their function [1]. It is caused by mutations in the CF transmembrane conductance regulator (*CFTR*) gene, encoding an important regulator for the transport of chloride and bicarbonate ions across epithelial cell membranes. Although there is no cure for CF, early diagnosis through newborn screening (NBS) combined with appropriate prompt treatment results in significant benefits for children born with CF [2,3]. These benefits outweigh risks of diagnostic uncertainty such as false-positive screening results. Therefore, NBS for CF is established in many countries or regions around the world and continues to be added to screening programmes [4]. 

The primary parameter is always immunoreactive trypsinogen (IRT) concentration in blood sampled as dried blood spots (DBS)s during the first days of life [5]. If positive (above cut-off), measurement of pancreatitis-associated protein (PAP) and/or DNA mutation analysis of the *CFTR* gene from the first card and a second card requested for a further IRT measurement are possible with different algorithms and procedures [6]. Thus, several strategies with numerous variances to screen newborns for CF exist. Importantly, a sweat test is used to confirm a diagnosis of CF for all screen-positive children [7].

In Austria, newborns have been screened for CF within the national NBS program since 1997 [8]. The Austrian NBS is centrally organized with a single laboratory at the Medical University of Vienna responsible for the analysis of about 85,000 newborns in Austria per year. Treatment and follow-up of affected patients are well organized routines in Austria, particularly for children and adolescents [9]. Until May 2017, IRT was the only parameter determined from DBSs. In the case of a positive result from the first card, a second card was obtained (IRT-IRT strategy) and a positive IRT value from this second card was referred to as “screen-positive”, resulting in a call for a sweat test for final diagnosis. Despite the high sensitivity (>95% on average), the positive predictive value (PPV) of this IRT-IRT algorithm was less than 15% and therefore markedly below an acceptable value [10]. Even more problematic were the high numbers of second cards (0.9%) requested using this algorithm, causing anxiety and stress in about 800 families in Austria per year.

The discovery of pancreatitis-associated protein (PAP) [11] as a suitable biomarker for second-tier analysis from the first DBSs as well as different genetic approaches for second or third-tier testing [12,13] opened new possibilities prompting us to review the CF screening algorithm in Austria. Due to ethical, legal, practical, and economic concerns [6,14,15], inclusion of genetic tests into the screening algorithm had a priori been excluded for further consideration. Therefore, an IRT-PAP-IRT algorithm including a fail-safe strategy and a two-step cut-off for PAP was adjusted from published variants [16] and implemented in the Austrian NBS in May 2017. Although widely published [16,17,18,19,20], a two-step IRT-dependent PAP cut-off is somewhat arbitrary and a more dynamic cut-off determination was sought. Weidler et al. showed that the IRT×PAP product showed better discrimination for classical CF than PAP alone as a second-tier screening parameter [21]. Therefore, after a 16-month evaluation period of the new algorithm, we combined these variants by including an IRT×PAP product cut-off into the scheme for the last 12 months of the observation period presented here. We compared performance parameters to other published algorithms, thereby showing that this relatively simple and economically inexpensive algorithm performs effectively.

## 2. Materials and Methods

### 2.1. Patients

**Figure 1:**
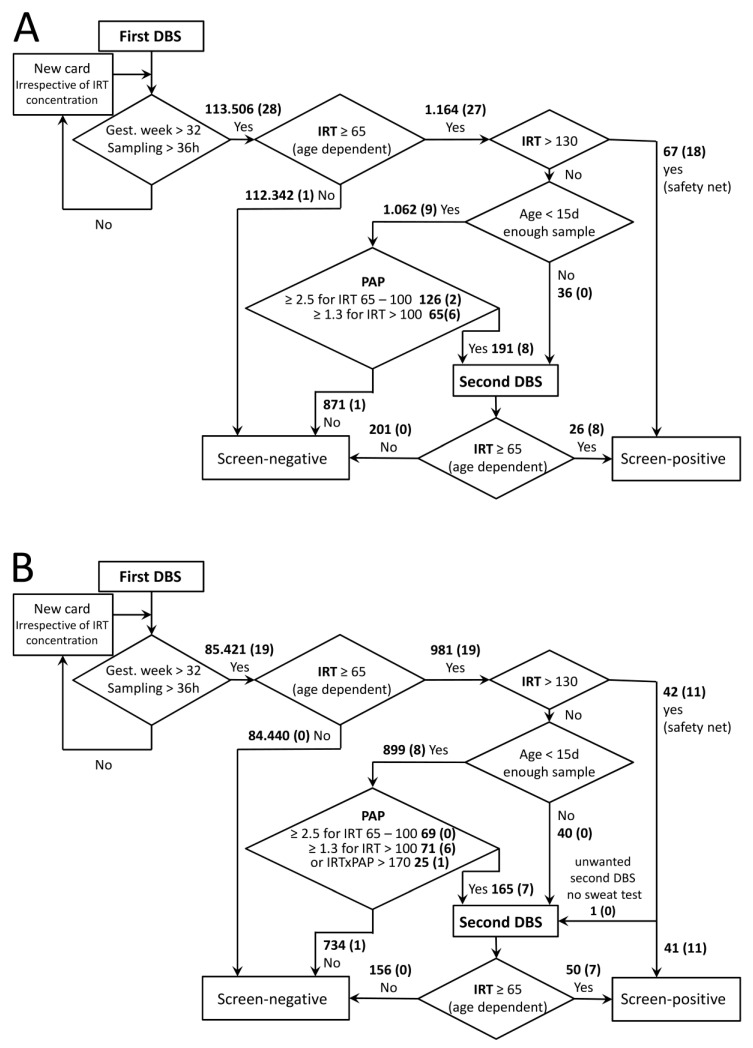
CF Screening schemes in Austria. Algorithms for the first period (**A**) and the second period (**B**) are depicted. Bold numbers give numbers of cards within indicated categories. The number in brackets is the number of confirmed positives within these categories. IRT and PAP cut-off concentrations are given in ng/mL, IRT × PAP in ng^2^/mL^2^.

In Austria, newborns are screened for CF as part of the regular NBS program. DBSs are recommended to be sampled between the 36th and 72nd hour of life and sent to the NBS laboratory located at the Department of Pediatrics and Adolescent Medicine of the Medical University of Vienna. If samples are taken before the 36th hour of life or before completed 32nd gestational week, a second card is requested and handled as a first card concerning CF screening irrespective of the initial result (Figure 1). The same accounts for cards of insufficient sampling quality or not enough blood. Within the observation period of May 2017 to August 2019, 198,927 DBS cards of newborns were received, which were suitable as first cards of the CF screening. This number corresponds to >99.5% of newborns born in Austria within this period.

### 2.2. DBS Tests

IRT blood concentrations were measured using the AutoDELFIA Neonatal IRT kit (Perkin-Elmer, Turku, Finland) according to manufacturer’s instructions. When IRT results were between 65 and 130 ng/mL and the newborn was less than 15 days old, PAP was measured as described below [22]. For children older than 14 days, measurement of samples with elevated IRT (age dependent cut-off values: up to 4 weeks of age, 65 ng/mL and; 5th to 6th week of life, 50 ng/mL; 7th to 9th week of life, 30 ng/mL) was repeated in duplicate. When the repeat analysis confirmed the raised IRT measurement, a second card was requested. If the initial result was above 130 ng/mL, measurement was also repeated in duplicate and if the mean exceeded the cut-off, patients were regarded as screen-positive. CF screening is repeated for all second cards requested for suspected biotinidase deficiency as low biotinidase activities can indicate sample deterioration due to heat and humidity [23]. 

**Figure 2:**
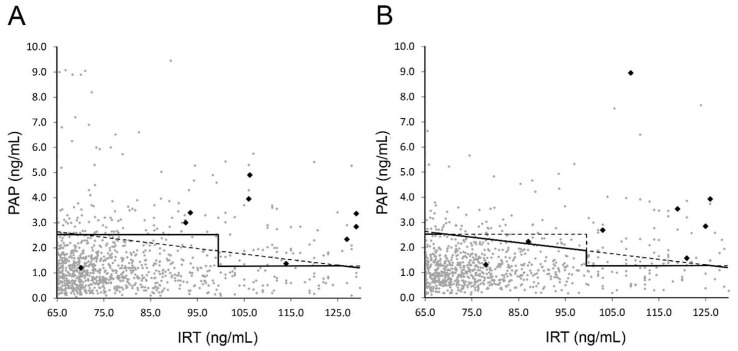
PAP vs. IRT values from first DBSs. Values of samples with IRT between 65 and 130 ng/mL eligible for PAP analysis according to the screening scheme for (**A**) first period and (**B**) second period are plotted. Solid lines indicate used cut-off values for request of second card; dashed lines give comparison to alternative strategies (see main text). Black data points indicate confirmed CF cases.

MucoPAPII (Dynabio, Marseille, France) was used to determine PAP blood concentrations in duplicate according to the manufacturer’s instructions. Cut-offs for PAP were ≥2.5 and ≥1.3 ng/mL for IRT 65–100 and 100–130 ng/mL, respectively. For comparison with earlier publications, these values are obtained with the MucoPAP II Kit, which was altered by the manufacturer in January 2017, resulting in a shift of values to 83% compared to the previous kit as determined by a series of comparisons in our laboratory. The PAP cut-offs were adjusted accordingly from previously published values [19]. In addition to these dual cut-offs, in the last 12 months of the observation period, a PAP×IRT cut-off [21] was introduced. To avoid a loss of sensitivity compared to the previous screening algorithm, values exceeding either of these cut-offs led to request of a second card. The PAP×IRT cut-off was set to 170 ng^2^/mL^2^, resulting in a smoothing of the step between the two PAP cut-off values, as depicted by the solid line in Figure 2B. PAP values rise with age, and age-dependent cut-offs are not available yet [22]. Therefore, for second cards, PAP was not measured and age-dependent cut-offs were applied for IRT as given above, and in the case of exceeding these values, patients were regarded as screen-positive.

### 2.3. CF Diagnosis

Parents of screen-positive newborns are referred to a pediatric CF center for a sweat test to be performed. A sweat chloride concentration above 60 mmol/L is considered CF-positive, leading to genetic testing and diagnostic, as well as therapeutic work up. Borderline sweat test results (30 to 60 mmol/L chloride) are followed by the diagnostic procedure recommended by the European CF Society [24]: Patients undergo a repeat sweat test and further evaluation in a specialist CF center, including detailed clinical assessment and extensive *CFTR* gene mutation analysis. In a first step, a targeted detection approach is applied using kits for multiplex allele-specific PCR amplification, which generates fluorescently labelled fragments that are analyzed by capillary electrophoresis. Used kits are, for instance, the CF-EU2 kit (Elucigene, Manchester, UK), identifying 50 mutations in total covering about 95% of mutations in Austria and the CFTR 68 kit (Devyse, Hagersten, Sweden) detecting 68 mutations. In case of inconclusive results, which means no or just one detected mutation, sequencing of the *CFTR* exon, and, if still inconclusive, the whole gene locus by next generation sequencing is performed. Additionally, structural variants are identified by multiplex ligation-dependent probe amplification. 

All results of sweat tests and most of the follow-up diagnostic results are reported to the NBS laboratory by the 10 CF centers in Austria. Since clinically diagnosed cases of CF are also routinely reported to the national NBS laboratory whatever their screening test result, the calculated sensitivity reported here has a high probability of accuracy. A small possibility exists that not all clinically diagnosed cases are notified to the national laboratory. Negative screen results for babies found clinically with meconium ileus are not classified as false-negatives.

## 3. Results

In the first period (16 months), 113,506 DBSs of newborns were screened for CF, 112,342 of which were initially screen-negative and not further considered.

Interestingly, a large proportion of screen-positives (67 out of 93) and the majority of confirmed positives (18 out of 28, for details of confirmed CF cases see Table 1) were found via the safety net (IRT above 130 ng/mL). Eight of the 9 other positive cases were detected via PAP analysis, which included 1062 (0.9%) samples with one false-negative (Figure 1A). This false-negative case with an IRT of 71 ng/mL and a PAP of 1.3 ng/mL (Figure 2A) was identified due to family history, but was a foreign citizen who left Austria after birth and therefore we do not have any further information. Sample quality of all false-negatives described here appeared sufficient not only in the receiving inspection but also in retrospective examination.

The majority of confirmed CF cases had an initial IRT value above 100 ng/mL. Only three confirmed positives, including the false-negative case, fell into the 65–100 ng/mL value category that accounted for 126 of the requested 191 second cards. Altogether, as the main performance indicators, a PPV of 29.2% and a sensitivity of 92.8% were determined (Table 2). A single patient was diagnosed with CF out of the screen-negative cases, resulting in a false-negative for IRT screening (Figure 1A). This false-negative case with an IRT of 56 was identified due to clinical symptoms of a period of a hypochloraemic alkalosis and a wheezy bronchitis; the sweat test was borderline and the genetic analysis revealed the N1303K and D110H mutations with pancreatic sufficiency. When patients were sorted according to their initial IRT values (Table 1), DF508 homozygotes were widely distributed and no conclusions on associations between distinct mutations and the quantities of IRT and PAP could be made. This would require a much higher number of cases.

In the second period, the determination of screen-positives and confirmed cases via the safety net occurred in similar proportions as in the first period (Figure 1 B, Table 2). In these 12 months, due to the additional IRT × PAP criterion, 25 (0.03%) more second cards and 3 more sweat tests (screen- positive) had to be requested. Notably, one out of the confirmed positive cases was detected due to the IRT×PAP criterion (Figure 1B) and would not have been detected via the previous algorithm (Figure 2B; ID1, Table 1). Again, one false-negative case occurred in the PAP-dependent branch of the screening algorithm: A patient with IRT of 78 ng/mL and PAP of 1.31 ng/mL (Figure 2B) was reported to us as CF-positive. The patient was detected due to family history including known CF cases and consanguinity. The sweat test was borderline, the genetic analysis revealed a homozygous 2789 + 5G > A mutation with pancreatic sufficiency. 

A comparison of the two periods and the previous screening based solely on IRT, as well as a hypothetical screening based only on an IRT×PAP cut-off value of 170 ng^2^/mL^2^, is given in Table 2. Data show that introducing PAP into screening markedly reduced numbers of second cards requested as well false-positive screening results, as sensitivity remained high. Differences between the strategies appear small, but in the first period one confirmed case would have remained undetected by the IRT×PAP only strategy, while in the second period one case would have been missed by IRT-PAP (Figure 2).

## 4. Discussion

This study shows how introducing a PAP measurement into an existing NBS algorithm using the conventional IRT-IRT protocol reduces the need to request second cards by more than 70%. The PPV raised by more than 50% without a substantial decrease in sensitivity due to the reduction of false-positives. Therefore, our CF screening now compared very satisfactorily to other CF screening strategies [10,25]. We also show that the inclusion of an IRT×PAP cut-off has the potential to further improve the screening yield. In the first period, one confirmed case would have remained undetected by the IRT×PAP only strategy. Therefore, we decided to combine the previous dual cut-off with the IRT×PAP criterion to reduce the probability of false-negatives. In our case, this strategy caused only minimally more second cards, and one additional confirmed case was found screen-positive due to the IRT×PAP(>170 ng^2^/mL^2^) cut-off.

Previous studies showed that concerning cost effectiveness, IRT-PAP protocols are superior to IRT-DNA protocols [6]. Another advantage of strategies like the Austrian one to determine the genetic background only after diagnosis by sweat chloride testing is that there is no ethically problematic carrier detection and less detection of CF screen-positive, inconclusive diagnosis (CFSPID) cases [26]. CFSPID is a common problem occurring with mutation analysis-based CF screening strategies. This has led to the widespread view that the individual’s phenotype rather than an equivocal genotype should be treated [27]. On the other hand, taking into account the successful reduction of CF rates by effective carrier screening strategies [28], a lack of carrier detection could also be regarded as disadvantageous depending on ethics varying from country to country. Patients with CFSPID may benefit from detection by NBS [29], although uncertainty remains challenging for families and caregivers and strategies for the follow-up of these patients are not commonly established yet [27]. Several European countries do not use DNA testing for CF screening [30], and neonatal genetic screening is often not adequately addressed in European laws [31]. In conditions with permissive laws and readily available financing, genetic analysis including F508del *CFTR* mutation analysis and next generation sequencing is definitely a reasonable choice to reduce second cards and screening (false-)positive, as exemplified by the Danish 3-Tier system [32], or the German and Dutch algorithms that also include analysis of PAP [19,33].

In conclusion, we here present a relatively simple and economical algorithm that performs effectively. This may be a useful model for NBS programs considering inclusion of CF into their screening panels or modification of existing schemes. However, our data on only a low number of confirmed positive cases also underline that there is still room for optimization of screening algorithms, ideally by multinational initiatives.

## Figures and Tables

**Table 1:** Newborn screening and genetic testing results of CF cases detected by the Austrian newborn screening within the overall observation period, sorted by initial IRT.

ID	IRT 1st DBS (ng/mL)	MucoPAP (ng/mL)	IRT 2nd DBS (ng/mL)	Mutation (Common Name)
1	87	2.2	73	DF508, homozygous
2	93	3.0	98	DF508/M1101
3	94	3.4	68	182delT/3849 + 10kbC->T
4	103	2.7	n.a.	DF508/R117H/7T9T
5	106	4.0	222	DF508, homozygous
6	106	4.9	246	DF508, homozygous
7	109	9.0	73	DF508, homozygous
8	114	1.3	n.a.	DF508, homozygous
9	119	3.5	250	DF508, homozygous
10	121	1.6	124	N1303K/R347P
11	125	2.8	113	DF 508/G542X
12	126	3.9	208	DF508/R117H/7T9T
13	127	2.3	80	S466X/R1070Q
14	129	2.8	181	DF508/G551D
15	136			DF508, homozygous
16	140			I807M, homozygous
17	140			DF 508/182delT
18	144			R553X/R117H/7T
19	144			DF508, homozygous
20	145			DF508, homozygous
21	152			DF508/R117H/7T9T
22	159			N1303K/Q39X
23	161			DF508, homozygous
24	165			S549N/*CFTR*50kbdel
25	172			DF508, homozygous
26	187			DF508, homozygous
27	191			DF508, homozygous
28	191			DF508/I507del
29	197			DF508l/R1162X
30	198			DF508, homozygous
31	200			DF508, homozygous
32	212			DF508/G542X
33	213			DF508/MET 82 Val
34	219			DF508, homozygous
35	226			DF508, homozygous
36	229			DF508, homozygous
37	234			DF508, homozygous
38	237			DF508, homozygous
39	246			DF508/R1162X
40	265			DF508/G542X
41	269			DF508, homozygous
42	307			DF508/G551D
43	330			DF508, homozygous
44	485			DF508, homozygous

**Table 2:** Comparison of different CF screening algorithms in Austrian newborn screening.

Algorithm	Second Cards	Safety Net	Screen- Positive	PPV	Sensitivity
Period 1 (IRT-PAP) *n* = 113.506	0.20% (227) ^1^	0.06% (67) ^1^	0.08% (93)	29.2% ^2^ (26/89)	92.8% (26/28)
Period 2 (IRT-PAP OR IRT×PAP) *n* = 85.421	0.24% 206)	0.05% (41)	0.11% (91)	22.2% ^3^ (18/81)	94.7% (18/19)
IRT-PAP only (hypothetical, calculated for period 2) *n* = 85.421	0.21% (181)	0.05% (41)	0.10% (88)	21.8% (17/78)	89.47% (17/19)
IRT×PAP only (hypothetical, calculated for period 2) *n* = 85.421	0.22% (191)	0.05% (41)	0.11% (90)	22.5% (18/80)	94.7% (18/19)
Previous Screening (IRT-IRT, 2 yrs) *n* = 172,322	0.88% (1519)	-	0.17% (285)	14% (36/285)	94.7% (36/38)

^1^ absolute numbers are given in brackets; ^2^ Four screen-positives have died, not included in PPV calculation; ^3^ Seven screen-positives have died, three lost to follow up, not included in PPV calculation.

## Data Availability

The data presented in this study are available on request from the corresponding author. The data are not publicly available due to privacy restrictions.
